# Chronic *Toxoplasma gondii* infection contributes to perineuronal nets impairment in the primary somatosensory cortex

**DOI:** 10.1186/s13071-022-05596-x

**Published:** 2022-12-24

**Authors:** Ramayana Morais de Medeiros Brito, Ywlliane da Silva Rodrigues Meurer, Jully Anne Lemos Batista, Andréa Lima de Sá, Cássio Ricardo de Medeiros Souza, Janeusa Trindade de Souto, Valter Ferreira de Andrade-Neto

**Affiliations:** 1 Postgraduate Program in Parasitary Biology, Department of Microbiology and Parasitology, Federal University of Rio Grande do Norte, Natal, Rio Grande do Norte Brazil; 2 Laboratory of Malaria and Toxoplasmosis Biology, Department of Microbiology and Parasitology, Federal University of Rio Grande do Norte, Natal, Rio Grande do Norte Brazil; 3 Postgraduate Program in Cognitive Neuroscience and Behavior, Memory and Cognition Studies Laboratory, Federal University of Paraíba, João Pessoa, Paraíba Brazil; 4 Laboratory of Immunopharmacology, Department of Microbiology and Parasitology, Federal University of Rio Grande do Norte, Natal, Rio Grande do Norte Brazil

**Keywords:** *Toxoplasma gondii*, Brain infection, Perineuronal nets, Primary somatosensory cortex, Inflammation

## Abstract

*Toxoplasma gondii* is able to manipulate the host immune system to establish a persistent and efficient infection, contributing to the development of brain abnormalities with behavioral repercussions. In this context, this work aimed to evaluate the effects of *T. gondii* infection on the systemic inflammatory response and structure of the primary somatosensory cortex (PSC). C57BL/6 and BALB/c mice were infected with *T. gondii* ME49 strain tissue cysts and accompanied for 30 days. After this period, levels of cytokines IFN-γ, IL-12, TNF-α and TGF-β were measured. After blood collection, mice were perfused and the brains were submitted to immunohistochemistry for perineuronal net (PNN) evaluation and cyst quantification. The results showed that C57BL/6 mice presented higher levels of TNF-α and IL-12, while the levels of TGF-β were similar between the two mouse lineages, associated with the elevated number of tissue cysts, with a higher occurrence of cysts in the posterior area of the PSC when compared to BALB/c mice, which presented a more homogeneous cyst distribution. Immunohistochemistry analysis revealed a greater loss of PNN labeling in C57BL/6 animals compared to BALB/c. These data raised a discussion about the ability of *T. gondii* to stimulate a systemic inflammatory response capable of indirectly interfering in the brain structure and function.

**Graphical Abstract:**

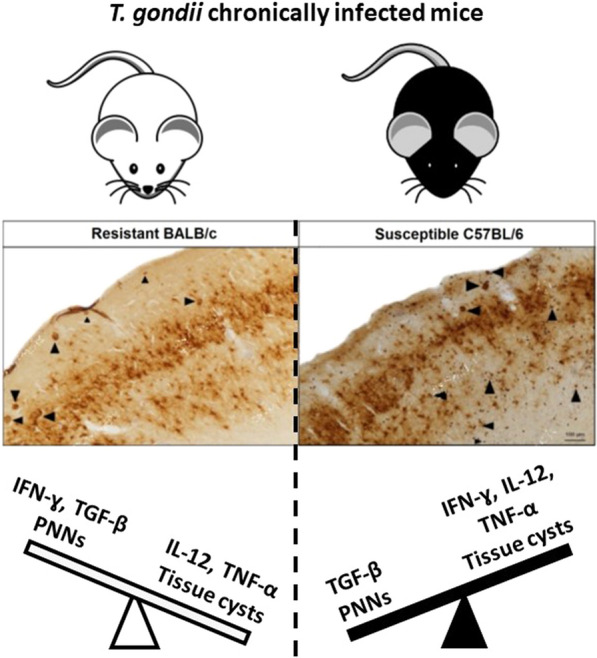

## Introduction

Toxoplasmosis is a zoonosis caused by the intracellular parasite *Toxoplasma gondii*. In the last years, the infection became the focus of discussion regarding behavioral changes among infected healthy individuals, with studies suggesting how the parasite could affect the cerebral homeostasis, developing a neuropathological state and establishing a correlation between the infection and cases of schizophrenia, bipolar disorder, aggression, impulsivity and depression [8, 9, 16, 18, 42, 30].

The ability of *T. gondii* to interfere in host behavior could be explained by interference in neurotransmitter production and metabolism [21, 37], the capacity to induce a neuroinflammatory response associated with the parasite’s arrival in the cerebral parenchyma and recruitment of circulating immune cells during systemic inflammation and production of inflammatory cytokines such as IFN-γ, IL-12 and TNF-α. Such cytokines are crucial for parasite control and chronic stage establishment; however, these cytokines can also contribute to neuronal damage [16, 36, 39].

The architecture of the central nervous system (CNS) is influenced by a highly specialized and complex extracellular matrix, described as perineuronal nets (PNNs), which surrounds specific neurons in the cortex areas, corpus striatum, hippocampus and spinal cord [7, 33, 44]. The role of these structural components is involved in the maintenance of neuronal circuitry [22], and its disruption increased the neuronal excitability. Such alterations could be linked to the development of neurological disorders [10, 12, 34].

The structure of PNNs is affected by many mechanisms, including enzymatic impairment due to the activity of matrix metalloproteinases (MMP) during trauma and/or inflammation. Also, PNNs' susceptibility to degeneration during inflammatory processes can contribute to synaptic impairment and neuronal plasticity in the CNS [22, 27, 36, 46]. However, there is still a lack of information regarding the possible role of *T. gondii* infection in stimulating the disruption of these structures.

In this context, this work aimed to investigate the effects of chronic *Toxoplasma gondii* infection on the integrity of PNNs in the primary somatosensory cortex (PSC) and the systemic cytokine profile of susceptible and resistant mice.

## Materials and methods

### Parasite and mouse infection

C57BL/6 and BALB/c mice, aged between 8 to 12 weeks old, were randomly distributed and housed in groups of five animals with ad libitum access to dry food and water, room temperature around 23 ± 2 °C and a 12-h light/dark cycle. This project followed the norms issued by the National Council for the Control of Animal Experimentation (CONCEA) and was approved by the Committee on Ethics in the Use of Animals of the Federal University of Rio Grande do Norte (CEUA/UFRN) under certificate number 124.048/2018.

For the experimental procedures, mice were orally infected with 25 cysts of *T. gondii* ME49 strain and followed for 30 days; during this period the weight loss and clinical signs were followed. The control mice were orally inoculated with sterile phosphate-buffered saline (PBS).

### Blood collection and enzyme-linked immunosorbent assay (ELISA)

After 30 days post-infection, the animals were killed with an overdose of xylazine and ketamine solution. Approximately 1 mL of blood was collected through cardiac puncture before the beginning of the perfusion protocol. The blood was kept at room temperature for 30 min for clot retraction, and then the serum was separated by centrifuging at 600 g for 10 min at 4 °C. The serum samples were kept at − 20 °C until use.

The quantification of TNF-α, IL-12, IFN-γ and TGF-β was performed by enzyme-linked immunosorbent assay using the ELISA Ready-SET-Go!^®^ (eBioscience™) kits following the manufacturer’s protocol. The samples were tested in triplicate with a dilution of 1:5 or 1:10, and the reaction was measured at 450 nm. The cytokine concentration was calculated using interpolation from a standard curve provided by each ELISA kit.

### Perfusion, brain removal and immunohistochemistry assay

Immediately after blood collection, the perfusion procedure was initiated with an intraventricular injection of 0.2 mL of the anticoagulant solution followed by transcardiac perfusion of 200 mL of 0.1 M phosphate buffer (PB) and 0.9% saline and 300 mL of paraformaldehyde 4% in 0.1 M PBS. After this procedure, the brains were carefully removed and kept in cryoprotectant 30% sucrose solution for 72 h at 4 °C. Sixty-μm-thick coronal sections were made in all brains and the sections were kept in the anti-freezing solution until the immunohistochemistry procedure.

Briefly, the brain sections were washed in 0.1 M PBS and then incubated with 0.3% hydrogen peroxide solution for 20 min. After washing in PB, the sections were incubated overnight with a 0.5% biotinylated *Wisteria floribunda* agglutinin (WFA, Sigma Co., USA), a lectin that can bind to acetylgalactosamine terminals of chondroitin sulfate proteoglycans in 0.1 M PBS + 0.3% Triton X-100. After washing, the sections were incubated with avidin-biotin-peroxidase complex (ABC kit, ThermoFisher Scientific™, USA) for 2 h. Following washing in PBS, the reaction was revealed after treatment of sections with 3, 3’-diaminobenzidine (DAB, Sigma Co., USA) solution in 0.1 M PBS for 5 min; 0.1 M PBS and 1% hydrogen peroxide were added to react for 5 additional min. Then, the sections were washed and mounted on gelatinized slides. After dehydration, the slides were coverslipped with Entellan^®^ and dried at room temperature for 5 days.

### Tissue cyst and cell quantification

Tissue cysts and WFA + cells were quantified in the PSC of each animal using an Olympus CX21 microscope with either × 10 or × 40 objectives. Six coronal sections per animal were used for analysis, with a distance of 120 μm between each section, and organized according to the anteroposterior orientation of the brain following the directions 0.50, − 0.70 and − 1.94 mm provided by the mouse brain stereotaxic atlas [19]. The WFA + cells were quantified considering the stained cell body and border. The tissue cysts were quantified according to their characteristic morphology. The images were made using an optical microscope (Nikon Eclipse Ni) with a digital video camera (Nikon DS-Ri) and objectives of × 4 and × 10. The images were minimally treated using the software GIMP 2.10.

### Statistical analysis

The results were expressed with boxplot using a minimum to maximum value plot to show the data. The normality and homoscedasticity of all data were analyzed by Shapiro-Wilk and Levene tests, respectively. Normal and/or homoscedastic data were analyzed with the ANOVA one-way test followed by Tukey’s post hoc test. Non-normal and/or homoscedastic data were analyzed using the Kruskall-Wallis test followed by Dunn’s test. For the tissue analysis, Mann-Whitney test was performed for non-parametric data and Student’s *t*-test for parametric data. Data were considered statistically significant when *P* < 0.05.

## Results

### Susceptible *Toxoplasma gondii*-infected mice exhibit higher levels of systemic inflammatory cytokines

**Fig. 1:**
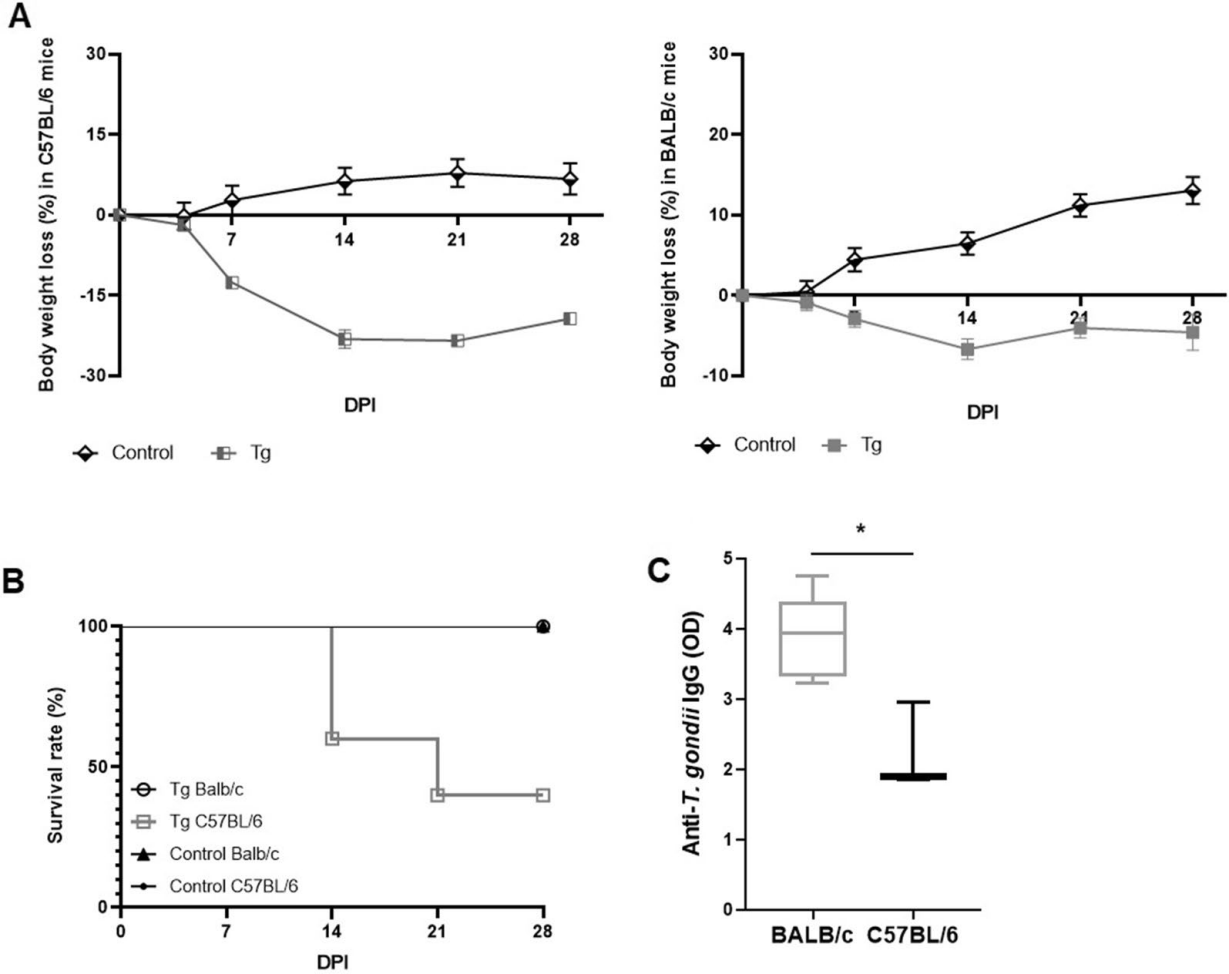
Evaluation of body weight loss, mortality rate and antibody production in susceptible and resistant mice infected with *Toxoplasma gondii*. **A** Body weight loss in susceptible C57BL/6 (left) and resistant BALB/c (right) mice infected with *T. gondii*. **B** Survival analysis of infected C57BL/6 and BALB/c mice during 30 dpi. **C** Quantification of specific anti-*T. gondii* IgG antibodies in the serum of mice. The survival rate was analyzed by the log-rank (Mantel-Cox) test followed by the Gehan-Breslow-Wilcoxon test. The antibody levels are expressed as optical density (OD). Five mice per group were used to perform the experimental protocol. Boxplot with minimum to maximum value plot was used to show the data. *Tg* group of mice infected with *T. gondii*, *DPI* days post-infection. *P < 0.05

Chronically infected mice displayed characteristic signs of acute infection, such as piloerection, and C57BL/6 exhibited higher weight loss and mortality than BALB/c mice (Fig. 1A, B). Regarding the antibody production, BALB/c displayed higher levels of specific anti-*T. gondii* systemic IgG antibodies than C57BL/6 mice (Fig. 1B).

**Fig. 2:**
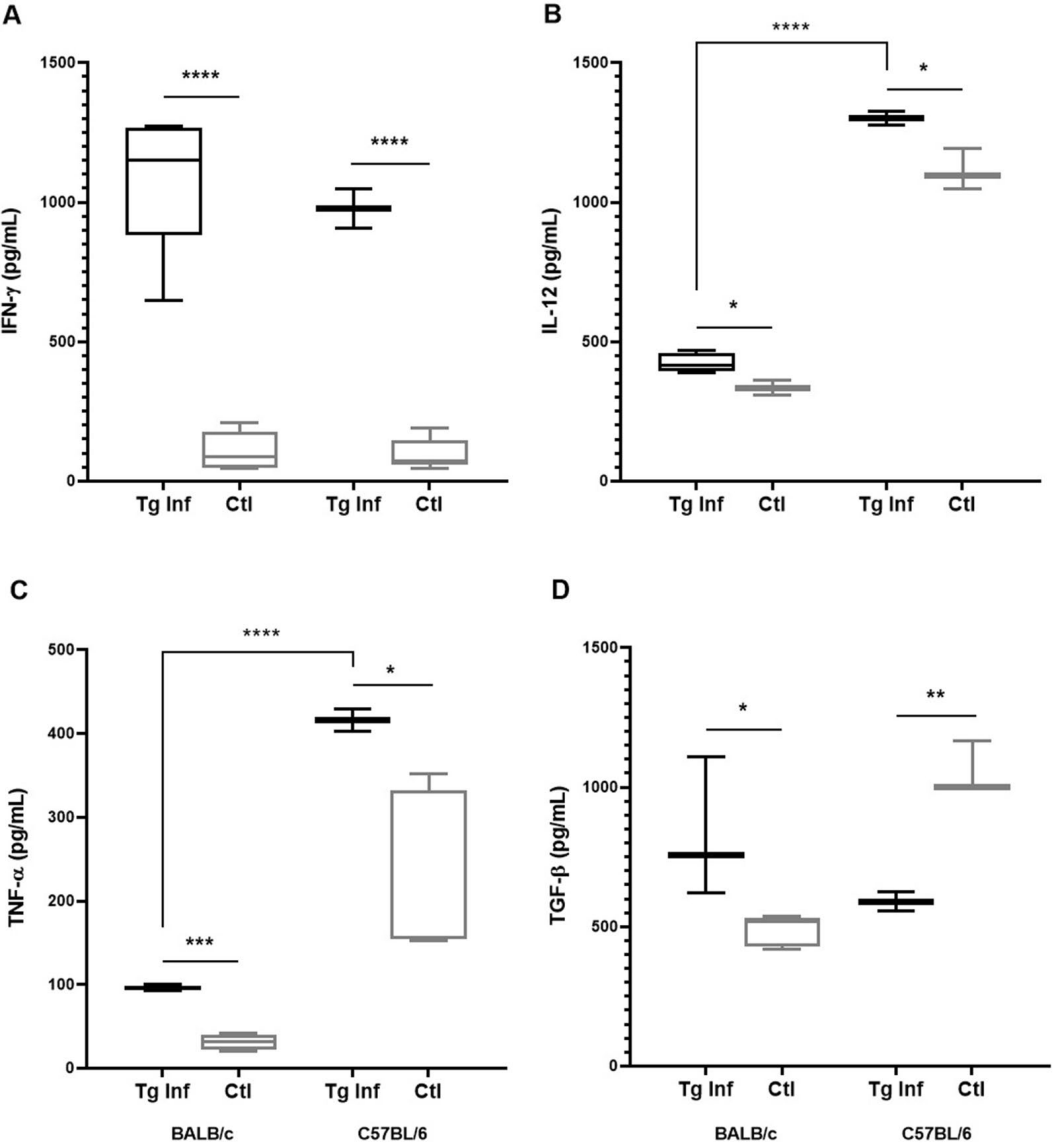
Quantification of systemic cytokine levels of resistant BALB/c and susceptible C57BL/6 mice chronically infected with *Toxoplasma gondii*. The systemic levels of IFN-γ (**A**), IL-12 (**B**), TNF-α (**C**) and TGF-β (**D**) in BALB/c and C57BL/6. Boxplot with minimum to maximum value plot was used to show the data. *P < 0.05; **P < 0.01; ***P < 0.001

The cytokine levels were determined in the serum obtained from infected and non-infected mice. Both infected groups revealed similar levels of IFN-γ (Fig. 2A), being detected as high as 1089.57 (± 256) pg/mL and 976.9 (± 101.2) pg/mL for infected BALB/c and C57BL/6 mice, respectively. These levels were significantly higher than those observed in control groups for both mice lineages (BALB/c Tg vs. control: 1089.84 ± 256 vs. 105.87 ± 70.34 pg/mL and C57BL/6 vs. control: 976.99 ± 101.2 vs. 96.22 ± 55.83 pg/mL, respectively).

Infected C57BL/6 exhibited higher levels of the cytokines IL-12 (Fig. 2B) and TNF-α (Fig. 2C) (1301.22 ± 36.08 and 415.7 ± 18.85 pg/mL, respectively) compared to infected BALB/c mice (422.9 ± 34.6 and 95.61 ± 3.17 pg/mL, respectively). The analysis of TGF-β levels revealed slightly higher titers for infected BALB/c mice (828.7 ± 251.4 pg/mL) than for infected C57BL/6 mice (590.26 ± 49.93 pg/mL); however, no significant differences were observed between the infected groups. The levels of TGF-β (Fig. 2D) were significantly higher than TNF-α and IL-12 in infected BALB/c mice (*P* < 0.0001; *P* = 0,0039, respectively); in the group of infected C57BL/6 mice, no difference was observed between TGF-β and TNF-α levels, and IFN-γ and IL-12 were significantly higher (*P* = 0.0102; *P* = 0.001, respectively) than TGF-β levels.

### Susceptible C57BL/6 mice presented a higher number of *Toxoplasma gondii* tissue cysts in the posterior area of the brain

**Fig. 3:**
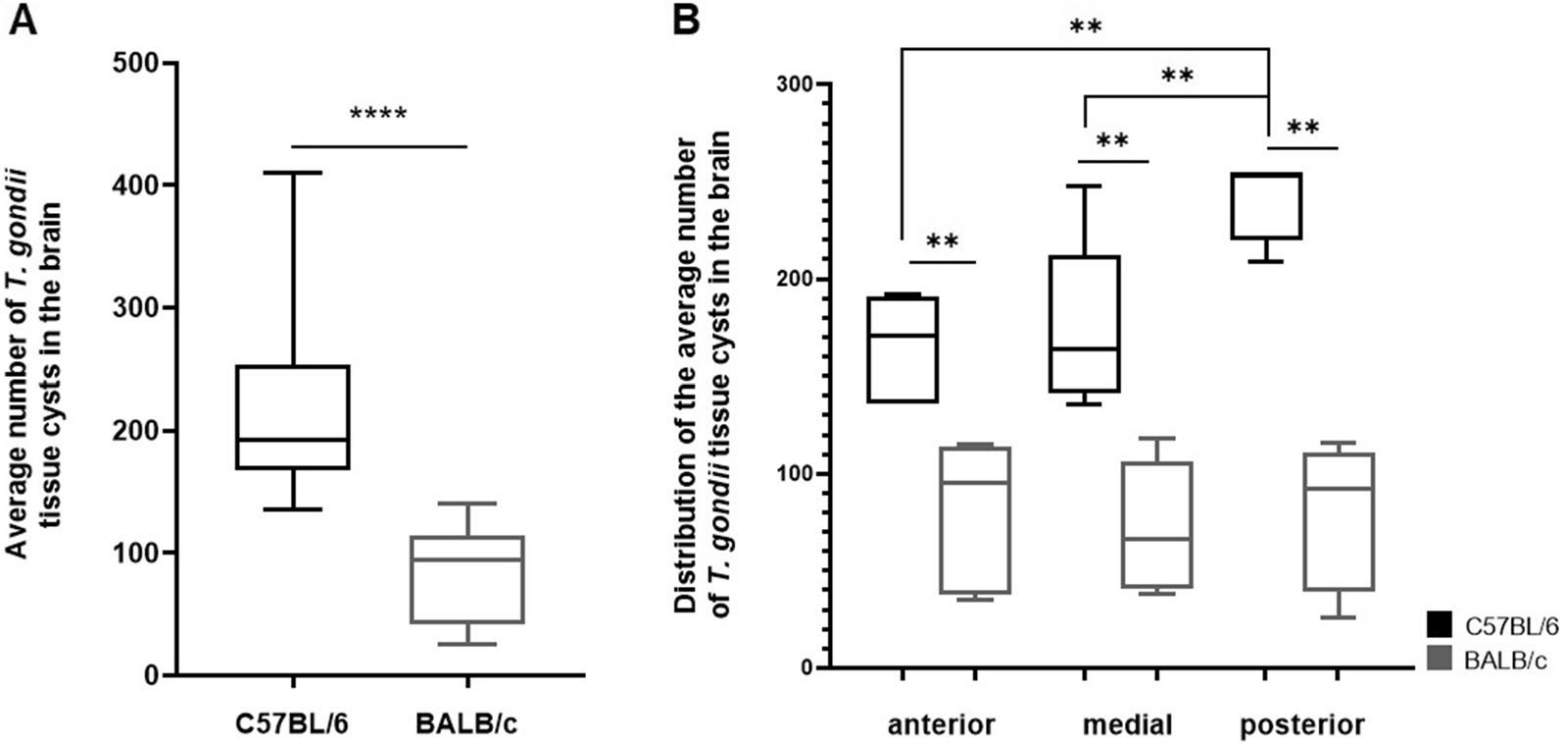
Quantification of *T. gondii* tissue cysts in the brain of chronically infected C57BL/6 and BALB/c mice. **A** Comparison of the average number of tissue cysts in the brain of infected C57BL/6 and BALB/c mice. **B** Distribution of tissue cysts in different portions of the brain in C57BL/6 (black) and BALB/c (gray). Boxplot with minimum to maximum value plot was used to show the data. **P < 0.01; ***P < 0.001

Analyzing the tissue cyst distribution between the infected mice lineages, it was observed that infected C57BL/6 mice exhibited a number of cysts 2.6 times higher than that observed for infected BALB/c mice (C57BL/6: 215 ± 71.74 cysts; BALB/c: 81 ± 36.49 cysts) (Fig. 3A). Aiming to verify the pattern of cyst distribution in the brain, it was observed that infected BALB/c mice showed a more homogeneous cyst distribution across the three analyzed regions (anterior: 79.6 ± 39.25; medial: 72 ± 33.82; posterior: 79.83 ± 36.52 cysts). On the other hand, infected C57BL/6 mice exhibited a less homogeneous distribution, with a predominant number of tissue cysts in the posterior area of the brain (anterior: 165 ± 27.7; medial: 174.2 ± 44.19; posterior: 242.5 ± 22.33 cysts) (Fig. 3B).

**Fig. 4:**
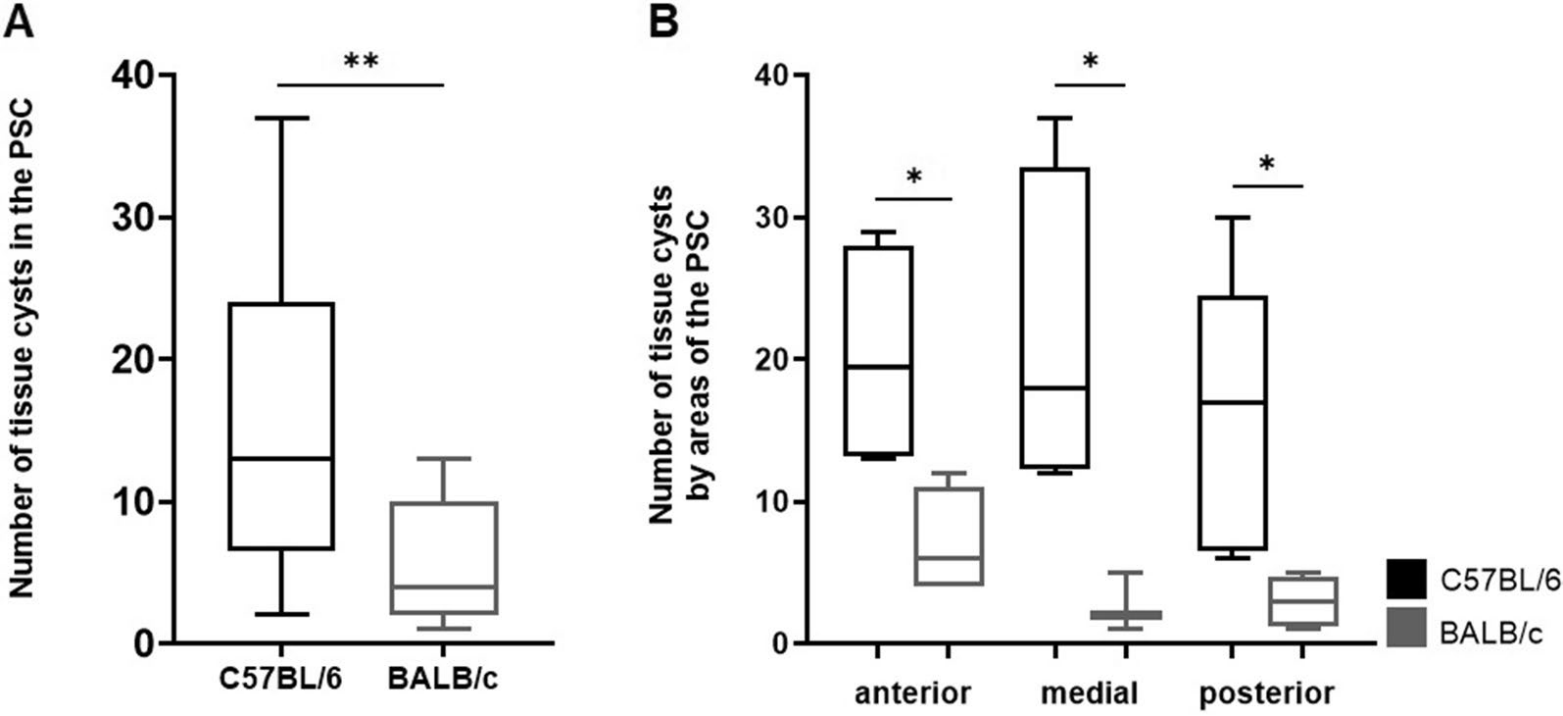
Quantification of *T. gondii* tissue cysts in the primary somatosensory cortex (PSC) of infected C57BL/6 and BALB/c mice. **A** Comparison of the average number of *T. gondii* tissue cysts in the PSC of infected C57BL/6 and BALB/c mice. **B** Analysis of the distribution of tissue cysts among different portions of PSC of infected C57BL/6 (black) and BALB/c (gray) mice. Boxplot with minimum to maximum value plot was used to show the data. *P < 0.05; **P < 0.01

Quantification of tissue cyst distribution in the PSC of both mice lineages revealed that infected C57BL/6 presented a number of tissue cysts three times higher than in infected BALB/c mice (C57BL/6: 15.51 ± 10.26 cysts, BALB/c: 5.61 ± 4.27 cysts) (Fig. 4A). Furthermore, significant differences in the number of tissue cysts were observed among the anterior (C57BL/6: 20.25 ± 7.97, BALB/c: 7 ± 3.83 cysts), medial (C57BL/6: 21.25 ± 11.61, BALB/c: 2.6 ± 2.08) and posterior (C57BL/6: 15.8 ± 9.83, BALB/c: 3.6 ± 1.52) portions of the analyzed area among the infected groups (Fig. 4B).

### Chronic infection led to a dramatic reduction of WFA + neuronal cells in the primary somatosensory cortex

**Fig. 5:**
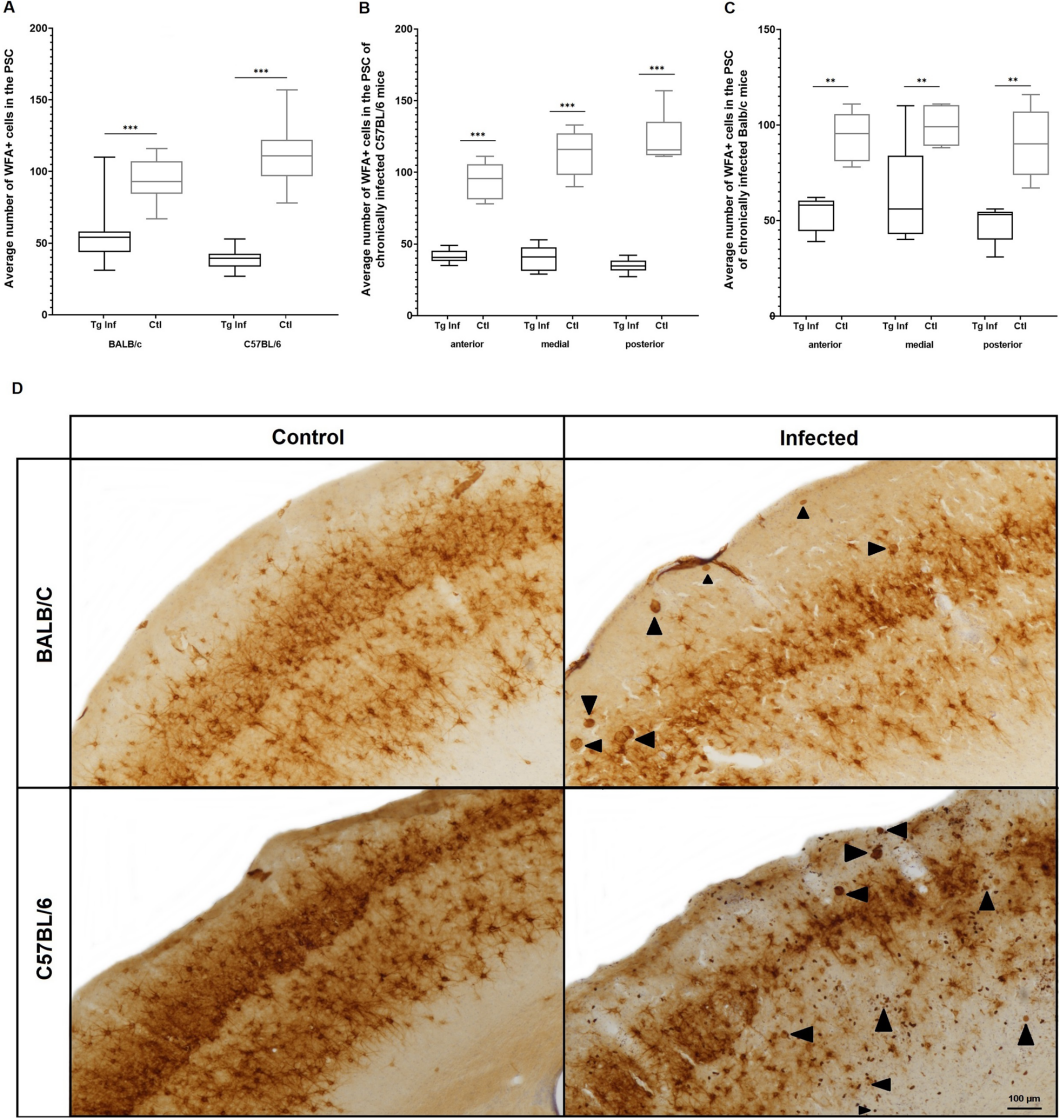
Quantification of WFA + neurons in the primary somatosensory cortex of *T. gondii* chronically infected susceptible and resistant mice. **A** Comparison of the average number of neurons labeled for WFA in the PSC of resistant (BALB/c) and susceptible (C57BL/6) mice chronically infected with *T. gondii*. **B** Distribution of WFA + neurons in the PSC of resistant C57BL/6 mice. **C** Distribution of WFA + neurons in the PSC of susceptible BALB/c mice. **D** Photomicrography showing the differences in the distribution of WFA + neurons in the PSC of chronically infected resistant BALB/c and susceptible C57BL/6 mice. Arrowheads indicate *T. gondii* tissue cysts, scale: 100 μm. Boxplot with minimum to maximum value plot was used to show the data. *P < 0.05; **P < 0.01; *** *P* < 0.001

Quantification of the total number of neuronal cells labeled for WFA in the PSC revealed a decrease of 42.4% and 65% of labeling in the infected BALB/c (54.25 ± 17.16 WFA + cells) and C57BL/6 mice (38.77 ± 6.78 WFA + cells), respectively, compared to the non-infected groups (BALB/c control group: 94.52 ± 13.94; C57BL/6 control group: 110.3 ± 19.16 WFA + cells) (Fig. 5A). The analysis of WFA + cells between the infected and control groups of each mouse lineage revealed a higher reduction in the labeling of WFA + cells for infected C57BL/6 mice in the anterior (41.33 ± 4.76), medial (40.33 ± 8.86) and posterior (34.66 ± 4.92) areas of the PSC (control group: 94.33 ± 12.69, 113.5 ± 16.02 and 123.16 ± 17.72 WFA + cells), representing a reduction of 56.18%, 64.46% and 71.85% of WFA + cells, respectively, while infected BALB/c mice displayed a reduction of 43.17%, 48.24% and 46.59% of WFA + cells in the anterior, medial and posterior areas of PSC (infected: 53.6 ± 9.28, 50 ± 8.48 and 48.33 ± 9.64 WFA + cells; control group: 94.33 ± 12.69, 99.6 ± 10.78 and 90.5 ± 17.96 WFA + cells for the anterior, medial and posterior areas, respectively) (Fig. 5B–C). The results presented here show that *T. gondii* chronic infection contributes to impairment of PNNs in the cortex of both susceptible and resistant mice, although susceptible mice display increased loss of PNN labeling (Fig. 5D).

## Discussion

The paradigm between susceptibility and resistance to *Toxoplasma gondii* infection can be explained by factors related to both host and parasite [1, 38, 49]. In our study, the differences observed during infection between the two mouse strains were evidenced by distinct weight loss and antibody production as well as mortality presented by susceptible C57BL/6 mice. Besides the increased weight loss in susceptible C57BL/6 mice, it was also found to have reduced levels of systemic anti-*T. gondii* IgG antibodies, which might be a reflection of the unbalanced inflammatory stimulation during *T. gondii* infection of susceptible mice.

In an inflammatory environment, the infection of susceptible C57BL/6 mice revealed production of IL-12 and TNF-α considerably higher than in BALB/c mice. Although the inflammatory response plays a key role in the establishment of an efficient anti-parasitic response, these inflammatory stimuli may contribute to the development of a severe immunopathology, compromising the mouse tissue integrity [20, 26, 48]. Here, the high levels of IFN-ɣ highlight its importance to the maintenance of a chronic infection, as this cytokine is shown to be crucial for the activation of astrocytes to control the parasite replication and cyst formation [24, 25]. In addition, it has been shown that high levels of TNF-α are related to increased macrophage infiltration and disruption of the blood-brain barrier (BBB) integrity [45] contributing to cerebral tissue damage. On the other hand, the production of TGF-β can interfere with the activation of inflammatory mediators, and the parasite can use the inhibitory activity to escape from a strong inflammatory response [51]. The role of TGF-β during *T. gondii* infection has been demonstrated by Cekanaviciute et al. [6], where inhibition of the TGF-β signaling pathway led to increased inflammatory cell infiltration, pro-inflammatory cytokine release and neuronal injury. Thus, the induction of TGF-β during *T. gondii* infection can be a crucial mechanism in preventing tissue damage by counterbalancing the proinflammatory pathway. In this context, the lack of balance during immune activation and failure to mount a protective response can contribute to parasite dissemination, followed by tissue damage, leading susceptible mice to succumb to infection.

To effectively invade the host cells, *Toxoplasma* can recognize abundant components of the extracellular matrix or widely distributed surface molecules, such as proteoglycans, specifically heparin and heparan sulfate and also chondroitin sulfate, as ligands for attachment to the host cell [5]. Regarding tissue cyst constituents, we understand that *T. gondii* bradyzoites reside, surrounded by a cyst wall rich in glycans, predominantly in brain and muscle tissue. The parasites are encased in a cyst matrix composed of several soluble components surrounded by a thick cyst wall. The cyst wall contains several prominent glycoproteins, including the major cyst wall glycoprotein CST1 (a 116 kDa glycoprotein). CST1 exhibits N-acetylgalactosamine moieties that are recognized by *Dolichos biflorus* agglutinin (DBA) as well as succinylated wheat germ agglutinin (s-WGA), which promotes staining of the cyst wall [23, 52]. In our work, tissue cyst identification in the brain was achieved considering the well-known morphological characteristics of *T. gondii* cysts. In addition, the protocol used here for labeling the PNNs was also able to evidence the cyst wall. Considering the presence of glycoproteins with acetylglucosamine terminals on the surface of the tissue cyst, it is possible that *Wisteria floribunda* agglutinin might recognize these structures, contributing to highlighting the limits of *T. gondii* cyst wall.

The resistance characteristic to infection with the ME49 strain of *T. gondii* exhibited by BALB/c mice can be exemplified by the significantly lower number of cysts observed when compared to C57BL/6 mice, associated with lower levels of TNF-α and IL-12, and higher levels of TGF-β. The comparison of susceptibility and resistance profile of C57BL/6 and BALB/c mice revealed an unbalanced inflammatory response in C57BL/6, which fails to control parasite replication and contributes to cerebral impairment with increased BBB breakdown [41]. Additionally, it has been shown that resistant mice have reduced levels of toxoplasmic encephalitis, which is represented by a lower number of inflammatory cells infiltrating and decreased levels of inflammatory cytokines [2]. Besides the random distribution of brain cysts, a significantly higher number of cysts was observed in the posterior portion of the brain in susceptible C57BL/6 mice. This finding can be related to the increased inflammatory infiltrate, which follows the main points of vascularization of the brain during early acute infection [40].

In addition to the cyst location in the brain, another interesting point highlighted in our study for the host-pathogen interaction is the ability of *T. gondii* to directly, or indirectly through systemic inflammation, contribute to the disruption of PNNs and thus compromise the cerebral integrity and circuitry functioning during chronic infection. In contrast to infected BALB/c mice, infected C57BL/6 showed a marked reduction of PNNs, which could be related to the higher systemic inflammatory levels observed among them. One of the main factors involved in the disruption of PNNs is the highly inflammatory environment, rich in reactive oxygen species, nitric oxide and activated matrix metalloproteinases [4, 14, 13, 32, 47, 50].

Inflammation contributes to the damage of cerebral parenchyma with disruption of extracellular matrix components, stimulating leukocyte migration to the infection sites [29]. Studies point to the possible preference of *T. gondii* for neurons and the further development of cysts in the neuronal projections far from the cellular body. This would stimulate the neuronal damage during chronic infection, visualized by the reduction of the neuronal number and increased apoptosis of these cells [3, 17, 43]. In addition, the neuronal loss shown by Li et al. [28] revealed that chronic *T. gondii* infection stimulated degeneration of cortical neurons, mainly GABAergic and glutamatergic neurons, with high levels of CX3CL1. It was also shown that chronic *T. gondii* infection contributes to phagocytic clearance of degenerating neurons through complement and activated microglia interactions [15, 28].

All the data presented here highlight the ability of *T. gondii* to interfere in the cerebral structure, which might be involved in the assembly of abnormal behavior as shown in our previous work, in which *T. gondii*-infected mice exhibited reduced PNNs surrounding neurons and displayed a hyperlocomor profile [11, 31]. In this context, and considering the differences in the inflammatory level among susceptible and resistant infected mice, it is plausible to establish a relationship between the immunological response triggered and modulated by *T. gondii* with the impairment of structural components of the CNS of susceptible C57BL/6 and resistant BALB/c mice.

## Data Availability

Not applicable.
